# The Generalizability of a Medication Administration Discrepancy Detection System: Quantitative Comparative Analysis

**DOI:** 10.2196/22031

**Published:** 2020-12-02

**Authors:** Eric Kirkendall, Hannah Huth, Benjamin Rauenbuehler, Adam Moses, Kristin Melton, Yizhao Ni

**Affiliations:** 1 Center for Healthcare Innovation Wake Forest School of Medicine Winston Salem, NC United States; 2 Department of Pediatrics Wake Forest School of Medicine Winston Salem, NC United States; 3 Department of Pediatrics University of Cincinnati College of Medicine Cincinnati, OH United States; 4 College of Medicine University of Tennessee Health Science Center Memphis, TN United States; 5 University of Iowa Iowa City, IA United States; 6 Department of Internal Medicine Wake Forest School of Medicine Winston Salem, NC United States; 7 Division of Neonatology and Pulmonary Biology Cincinnati Children’s Hospital Medical Center Cincinnati, OH United States; 8 Division of Biomedical Informatics Cincinnati Children's Hospital Medical Center Cincinnati, OH United States

**Keywords:** medication administration, error, automated algorithm, generalizability, quantitative comparative analysis, discrepancy, detection, quantitative analysis, portability, performance algorithm, electronic health record

## Abstract

**Background:**

As a result of the overwhelming proportion of medication errors occurring each year, there has been an increased focus on developing medication error prevention strategies. Recent advances in electronic health record (EHR) technologies allow institutions the opportunity to identify medication administration error events in real time through computerized algorithms. MED.Safe, a software package comprising medication discrepancy detection algorithms, was developed to meet this need by performing an automated comparison of medication orders to medication administration records (MARs). In order to demonstrate generalizability in other care settings, software such as this must be tested and validated in settings distinct from the development site.

**Objective:**

The purpose of this study is to determine the portability and generalizability of the MED.Safe software at a second site by assessing the performance and fit of the algorithms through comparison of discrepancy rates and other metrics across institutions.

**Methods:**

The MED.Safe software package was executed on medication use data from the implementation site to generate prescribing ratios and discrepancy rates. A retrospective analysis of medication prescribing and documentation patterns was then performed on the results and compared to those from the development site to determine the algorithmic performance and fit. Variance in performance from the development site was further explored and characterized.

**Results:**

Compared to the development site, the implementation site had lower audit/order ratios and higher MAR/(order + audit) ratios. The discrepancy rates on the implementation site were consistently higher than those from the development site. Three drivers for the higher discrepancy rates were alternative clinical workflow using orders with dosing ranges; a data extract, transfer, and load issue causing modified order data to overwrite original order values in the EHRs; and delayed EHR documentation of verbal orders. Opportunities for improvement were identified and applied using a software update, which decreased false-positive discrepancies and improved overall fit.

**Conclusions:**

The execution of MED.Safe at a second site was feasible and effective in the detection of medication administration discrepancies. A comparison of medication ordering, administration, and discrepancy rates identified areas where MED.Safe could be improved through customization. One modification of MED.Safe through deployment of a software update improved the overall algorithmic fit at the implementation site. More flexible customizations to accommodate different clinical practice patterns could improve MED.Safe’s fit at new sites.

## Introduction

Patient safety is maximized when medical errors are efficiently detected and mitigated or prevented in the first place. The most common type of medical errors are medication errors, which are defined as any preventable event that may cause or lead to inappropriate medication use or patient harm while the medication is in the control of the health care professional, patient, or consumer [1]. Medication errors can occur at all stages in the patient care process including ordering, transcribing, dispensing, administration, and monitoring [2-4]. In recent years, medication administration has been identified as an error-prone stage in the patient care process and comprises a large percentage of all medical errors [3]. Despite extensive efforts, medication administration errors (MAEs) continue to inundate patient care [5,6].

The persistence of medication errors has led to a need for clinical informatics methods and technological interventions to improve medication error detection and prevention [7,8]. Common informatics approaches to prevent errors include the use of dedicated systems such as clinical decision support during medication ordering in the electronic health record (EHR) or drug error reduction systems contained in smart infusion pumps; both provide overdose and other types of alerts [9,10]. The former system works to detect errors and reduce the total number of medication errors early in the medication use process (at the ordering stage) [11], but does not detect error types that are introduced downstream in the later phases such as medication administration. Improved efforts to detect different error types during the administration and monitoring phases can serve to capture issues that have propagated from early stages—in the event they are not already addressed by upstream systems—as well as detecting errors introduced later in the system [12]. By effectively detecting and identifying errors at any point of the medication use life cycle, it is possible to inform intervention and prevention strategies to prevent future errors of the same type and possibly mitigate harm [13-17].

The availability of digitized EHRs and medication administration records (MARs) make it possible to perform algorithmic analysis of the data to detect MAEs quickly and efficiently [12,14,18,19]. Furthermore, the EHR and the creation of care-related data afford the ability to detect MAEs or discrepancies across entire populations and large data sets. This is in contrast to current methods of detection, which usually rely on sampling strategies followed by selective manual review of records or by reviewing the output from voluntary reporting [13,15-17]. In our prior work [12,20-22], discrepancies were identified when an algorithm detected a difference between the dosage intended to be delivered (prescriber’s orders) and how it was documented as being delivered (MAR data). A dosing-related MAE was defined as any discrepancy between the medication dose or infusion rate administered to a patient and the dose/rate prescribed by physicians during patient care. However, a discrepancy only becomes an error when it is clinically valid and has the potential to cause harm to the patient. As a result, error rates (ie, clinically valid errors) and discrepancy rates (ie, algorithm-based detections) are not completely synonymous; high discrepancy rates do not directly correspond to high error rates or indicate suboptimal practice until the discrepancy is investigated and deemed an actual error. However, discrepancies give reviewers a starting point to efficiently find actual errors.

In this study, we sought to implement MED.Safe, a software package of medication discrepancy detection algorithms, and benchmark the results to our earlier work at the development site to determine its portability and generalizability. We analyzed the system outputs at an external site, highlighting where and in what context the system performed well, and suggested customizations to further improve its performance. This analysis will provide the basis for further implementation and scaling of the current software package into other health care institutions.

## Methods

### Study Setting

The study took place at Wake Forest Baptist Medical Center (WFBMC), a tertiary level 1 trauma center and level 1 pediatric trauma center with 885 beds in Winston-Salem, North Carolina. WFBMC implemented an EHR system (Epic Systems) in 2012. This study focuses on the pediatric intensive care unit (PICU) with 12 beds, the neonatal ICU (NICU) with 40 beds, and the adult medical ICU with 172 beds.

### Data Sources

Order and MAR data were extracted from the EHR for 11 medications prescribed at WFBMC: dobutamine, dopamine, epinephrine, fentanyl, insulin, intravenous (IV) fluids, lipids, milrinone, morphine, total parenteral nutrition (TPN), and vasopressin. The medications were originally selected by the investigative team (EK, KM, YN) because they were the continuously infused medications associated with the highest harm in the NICU setting. Structural differences in the format of 2 of the medication orders between the sites were taken into account during data extraction. At Cincinnati Children’s Hospital Medical Center (CCHMC), all TPN and IV fluids are contained in orders under 1 parent order for each medication/fluid category. At WFBMC, there is no single parent order, and additional mapping of the individual fluid and TPN orders was necessary. After accounting for this difference, the data from WFBMC were retrospectively extracted for the calendar year 2018 (January 1, 2018, to December 31, 2018). To compare system outputs, NICU data from CCHMC were also retrospectively extracted over the same period.

### MED.Safe System

MED.Safe is an automated software package that analyzes medication use information in EHRs to identify medication administration discrepancies [12,20,21]. The MED.Safe package was originally developed by CCHMC with the purpose of monitoring high-risk IV medications in the NICU setting.

The analyzed information includes (1) medication orders that document medication doses (or infusion rates) prescribed to the patients, (2) structured order modifications (audits) that adjust the original doses/rates via computerized physician order entry, (3) MARs that document actual doses/rates administered to patients, and (4) free-text physician to nurse communication orders that deliver complex dose/rate adjustment during patient care. The free-text communications were parsed with a set of regular expression–based natural language processing algorithms to identify discrete dose/rate changes. The output consists of matching ordered medication doses with those recorded on the MAR in chronologic order. Using the extracted information, the detector module identifies discrepant doses/rates between MARs and other data sources using a set of logic-based rules. The detector was built upon our earlier research on MAE detection, where the logic-based rules were abstracted from standard care practices, refined by neonatologists, and implemented by programmers. By analyzing the dynamic EHR information, the detector determines the latest dose/rate prescribed to a patient and matches it with an MAR dose/rate to determine whether a match or discrepancy is present. MED.Safe allows users to map data elements required by the computerized algorithms to the site’s EHR instance data model. Once the mapping is complete, MED.Safe automatically extracts data from the EHR instance, executes the discrepancy detection algorithms, and visualizes chronological ordering of the medication use data and the identified discrepancies (if any). It also generates descriptive statistics of the medication use data including numbers of orders, audits, MARs, and discrepancies for the studied medications.

### Study Design

**Figure 1:**
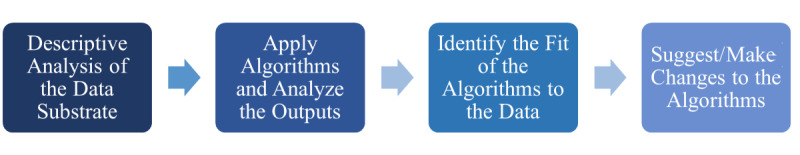
The overall processes of the study, for executing MED.Safe at a second site.

The investigative team (EK, BR, AM) executed the MED.Safe software package developed at CCHMC on the local WFBMC EHR data followed by a rigorous analysis of algorithm outputs. This step was completed entirely at WFBMC with guidance from the CCHMC study team (KM and YN). Analysis of the outputs was performed with the intent of learning the context within which the discrepancy detection algorithms were a good “fit” and performed accurately, and where they seemed to be inaccurate and needed customization for the new clinical environment. Figure 1 presents an overview of the study, and the individual methodological steps are further described in the following sections.

#### Phase 1: Analysis of WFBMC’s Medication Ordering Environment

To determine the fit and feasibility of MED.Safe at WFBMC, the investigative team (all study authors) analyzed the quantity and distribution of medication use data available. Descriptive statistics on medication orders, order modifications (ie, audits), and MARs generated by MED.Safe were aggregated by department (NICU, PICU, and adult medical ICU) and medication to study prescriber preferences and workflows. The analyzed MARs were restricted to actions including new bag, start, restart, rate verify, and rate change, to include administrations where potential administration errors could occur. Ratios comparing the numbers of audits, orders, and MARs were calculated for all ICUs at WFBMC and the NICU at CCHMC. The audit/order ratio represented the average number of times an order was modified during its life cycle, which implied prescribing patterns in a clinical environment (if prescribers frequently changed an order or kept a more stable prescribing habit). The MAR/(order + audit) ratio represented the average number of MARs documented by clinicians for each order or order modification, which suggested documentation patterns in a clinical unit.

#### Phase 2: Analysis of the MED.Safe Outputs to the Data From Another EHR Instance at WFBMC

After data element configuration, MED.Safe was executed against WFBMC’s clinical data repository to extract medication use data retrospectively. MED.Safe’s discrepancy detection algorithms were then performed for each WFBMC ICU department. We analyzed the results aggregated across the ICU departments and for WFBMC NICU solely and compared them with those from the development site (CCHMC) to determine specific settings (medications and clinical departments) that demonstrated the best fit and areas of improvement needed for the system. Results were visualized numerically and graphically to compare trends in discrepancy rates between WFBMC and CCHMC.

#### Phase 3: Analysis of System Generalizability and Areas of Improvement

We assumed that good system generalizability to the WFBMC data would be expected to yield discrepancy rates similar to the baseline rates at CCHMC. Discrepancy rates substantially higher than the baselines were assumed to indicate a poor fit, which prompted further investigation to confirm this assumption and suggest areas of improvement.

If the discrepancy rate for a medication was higher than expected compared to the baseline, the system outputs were inspected manually to identify potential causes. The numbers of processed medication orders, audits, and MARs were interrogated to understand and examine the possible effect of local medication use patterns. For example, a specific type of order or MAR entry triggering discrepancies on more than 1 occasion might indicate a pattern of interest. These patterns were investigated, and the inspection was completed for each medication.

#### Phase 4: Suggested Customization of the System to Enable Better Detection of Medication Administration Errors

Manual analysis of the patterns identified in phase 3 was completed by the investigative team (all study authors) to pinpoint whether the source of discrepancy deviation was technical (caused by algorithm logic) or a result of clinical factors (a change of prescribing practices between sites that the system was not capable of capturing), in order to improve accuracy in MAE detection.

The technical barriers to good fit that were identified were addressed through the addition of a software update where feasible. The updated system was then re-executed on the same 2018 WFBMC data set. The updated system outputs were compared to the original system outputs in terms of order counts, order audit counts, MAR counts, and discrepancy rates to understand the impact of the customizations.

## Results

### Phase 1: Analysis of WFBMC’s Medication Ordering Environment

Table 1 presents the distribution of medical use data for each ICU department at WFBMC. A total of 10,304 orders, 2647 audits, and 268,446 MARs were created during the study period. The NICU placed the most orders, made the most order modifications (audits), and created the most MAR entries. By contrast, the adult medical ICU had the least in all 3 categories, reflecting the fact that the MED.Safe system was originally designed for a pediatric population (the CCHMC NICU). Multimedia Appendices 1 and 2 present more specific breakdowns by medication and department, which suggested that IV fluids, TPN, lipids, and fentanyl were the most ordered medications and had the highest MARs in each of the investigated departments. The WFBMC NICU was the only investigated department without use of vasopressin and morphine; the other departments had orders and subsequent audits and MARs for all 11 medications studied. Additionally, the WFBMC NICU had almost 3 times the number of MARs when compared to the CCHMC NICU despite having only about half as many orders and audits. This was found to be the result of a practice of documenting rate verifications on the MAR much more frequently than the practice in the CCHMC NICU.

**Figure 2:**
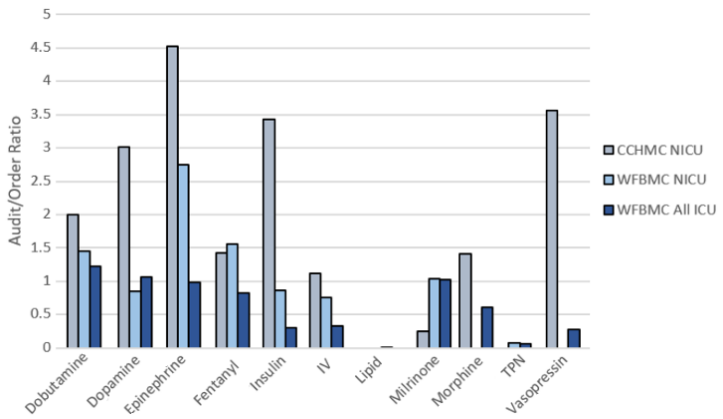
Comparison of audit/order ratios between (A) CCHMC NICU, (B) WFBMC NICU, and (C) WFBMC All ICUs. CCHMC: Cincinnati Children’s Hospital Medical Center; ICU: intensive care unit; NICU: neonatal intensive care unit; WFBMC: Wake Forest Baptist Medical Center.

The audit/order and MAR/(order + audit) ratios are presented in Multimedia Appendices 3-5 to compare the differences in prescribing habits and order fluidity between WFBMC and CCHMC. Figure 2 compares the audit/order ratios between all WFBMC ICUs, WFBMC NICU (NICU subset of all WFBMC ICUs), and CCHMC NICU. The ratios differed substantially between the 3 data sets across the studied medications. The CCHMC NICU had higher audit/order ratios for 7 of the 11 medications. For example, dopamine at CCHMC had an audit/order ratio of 3.0, whereas that medication at WFBMC had an audit/order ratio of 0.9.

**Table 1:** Distribution of medication orders, audits, and medication administration records in the WFBMC ICUs compared to the CCHMC NICU.

Distribution of data elements	WFBMC^a^ adult medical ICU^b^	WFBMC PICU^c^	WFBMC NICU^d^	CCHMC^e^ NICU
Number of orders	1950	1964	6390	12,603
Number of audits	576	934	1137	4386
Number of MARs^f^	38,787	62,780	166,879	56,715

^a^WFBMC: Wake Forest Baptist Medical Center.

^b^ICU: intensive care unit.

^c^PICU: pediatric intensive care unit.

^d^NICU: neonatal intensive care unit.

^e^CCHMC: Cincinnati Children’s Hospital Medical Center.

^f^MAR: medication administration record.

**Figure 3:**
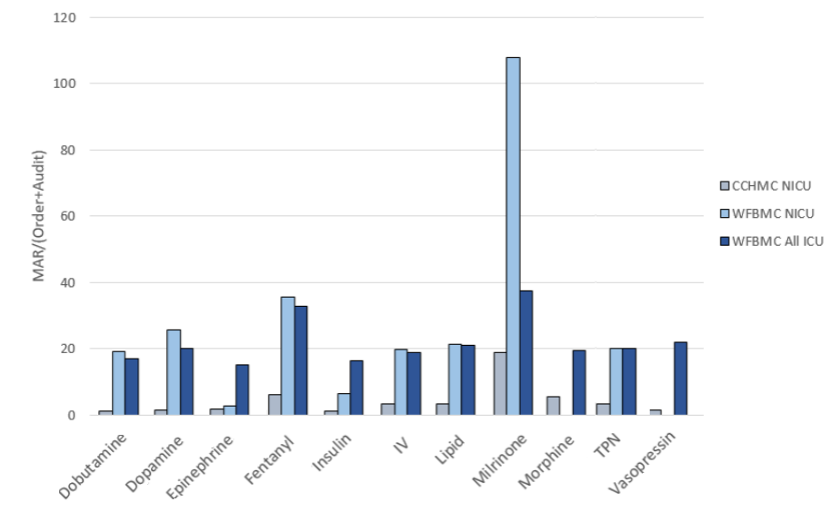
Comparison of MAR/(order + audit) ratios between the CCHMC NICU, the WFBMC NICU, and WFBMC All ICUs. CCHMC: Cincinnati Children’s Hospital Medical Center; ICU: intensive care unit; MAR: medication administration record; NICU: neonatal intensive care unit; WFBMC: Wake Forest Baptist Medical Center.

Figure 3 and Multimedia Appendices 3-5 present MAR/(order + audit) ratios between WFBMC departments and CCHMC NICU. The WFBMC NICU and all ICUs at WFBMC had comparable ratios. When compared to the CCHMC NICU, the ratios for WFBMC were higher for each studied medication. The average ratio for WFBMC NICU was 23.6 and the average for CCHMC was 4.4. The MAR/(order + audit) ratio for milrinone in the WFBMC NICU was higher than the other medications and departments. This is a result of WFBMC NICU’s practice to verify the rate of milrinone approximately every hour for the entire duration of the medication.

### Phase 2: Comparison of the MED.Safe Outputs to the Data From Another EHR Instance at the Second Site

Table 2 presents the discrepancy rate output by MED.Safe for each studied medication. Compared to the baseline discrepancy rates from CCHMC NICU, 5 out of 9 medications used at WFBMC NICU (excluding vasopressin and morphine that did not have orders) showed close discrepancy rates, with less than 1% difference. Epinephrine had similar discrepancy rates, with less than 3% difference. However, the discrepancy rates for insulin, dobutamine, and dopamine were exceptionally large, with over 5% difference. Compared to WFBMC NICU, the discrepancy rates at all WFBMC ICUs tended to deviate more from CCHMC NICU.

**Table 2:** A comparison of medication administration discrepancy rates generated by MED.Safe at Wake Forest Baptist Medical Center and Cincinnati Children’s Hospital Medical Center during the study period.

Medication	Discrepancy rate at all ICUs^a^ in WFBMC^b^, %	Discrepancy rate at NICU^c^ in WFBMC, %	Discrepancy rate at NICU in CCHMC^d^, %
Dobutamine	7.9	19.8	0.0
Dopamine	6.7	6.0	0.9
Epinephrine	20.9	4.7	2.1
Fentanyl	5.9	0.5	0.3
Insulin	41.7	59.3	4.3
Intravenous fluids	1.1	1.7	2.5
Lipids	0.1	0.0	0.1
Milrinone	1.1	0.3	0.0
Morphine	6.7	N/A^e^	0.1
Total parenteral nutrition	1.4	1.4	1.3
Vasopressin	2.1	N/A	2.3

^a^ICU: intensive care unit.

^b^WFBMC: Wake Forest Baptist Medical Center.

^c^NICU: neonatal intensive care unit.

^d^CCHMC: Cincinnati Children’s Hospital Medical Center.

^e^N/A: not applicable. In 2018, no orders for continuous morphine or vasopressin were placed in the WFBMC NICU.

**Figure 4:**
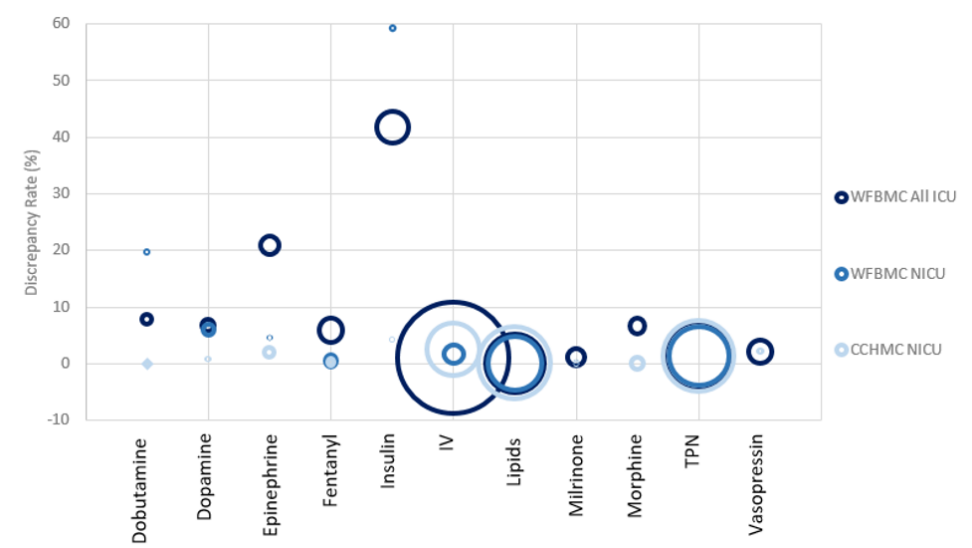
A comparison of discrepancy rates by medication and number of orders between (A) WFBMC All ICUs, (B) WFBMC NICU, and (C) CCHMC NICU. Circle radius correlates with the number of medication orders for the sites. CCHMC: Cincinnati Children’s Hospital Medical Center; ICU: intensive care unit; NICU: neonatal intensive care unit; WFBMC: Wake Forest Baptist Medical Center.

Figure 4 further depicts the relationship between site, discrepancy rate, and medication. A circle size represents the number of orders for a medication during the study period while plotting the discrepancy rate by medication and institutional site. For nearly all medications, the CCHMC NICU had lower discrepancy rates when compared to WFBMC sites and a larger number of orders when compared to the WFBMC NICU specifically. We observed that the outliers in discrepancy rates (epinephrine, dopamine, dobutamine, and insulin) were often due to a small number of orders as represented by the small circle radius.

### Phase 3: Analysis of System Generalizability and Areas of Improvement

We further investigated the medications with discrepancy rates that substantially deviated from the CCHMC baseline. Three primary causes for the deviation of discrepancy rates were identified: (1) range-based dosing (a common prescribing practice); (2) a data extraction, transforming, and loading issue causing initial order values to be overwritten in the data (a technical data processing issue); and (3) verbal ordering practices (site-specific prescribing practice).

**Figure 5:**
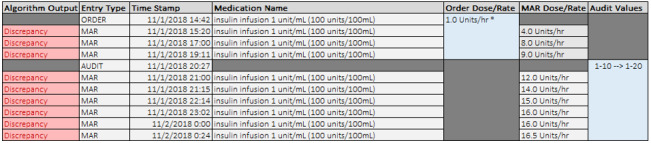
Example of a dosing range order interpretation issue by the algorithm. In this example, orders placed with dosing ranges are not interpreted correctly by the system in place to detect medical administration discrepancies. The algorithms, in their current state, do not expect a dosing range and mark the MAR as a discrepancy if the value doesn’t match the first value in the order dose range. Subsequent titrations that would fall within the acceptable range of the order are erroneously identified as discrepancies by the algorithm. *The Order Dose/Rate in this figure represents the value that the algorithm parses from the original order. In the instance of orders being placed with a dose range (ie, 1-10 Units/hr), the algorithms only parse and use the first value of the dose range. MAR: medication administration record.

At WFBMC, some medication orders are written as a dosing range (eg, insulin 1-10 Units/hr, with an associated titration protocol) rather than as a discrete dose (eg, insulin 1 Units/hr, titrate by 0.5 Units/hr). Because MED.Safe expects a determinate dose for high-risk IV medications per guidelines at CCHMC, the dosing range practice resulted in very high levels of discrepancies for some medications (eg, insulin) at WFBMC, as seen in Table 2. Figure 5 demonstrates an example system output for an order with a dosing range, including the order, audit, and MARs for a single patient spanning 2 calendar days. After reviewing the patient chart, it was discovered that the original order in the EHR was set to a range of 1-10 Units/hr and was changed to 1-20 Units/hr approximately 6 hours later. However, the MED.Safe system expected a discrete dose for insulin and converted the dosing range to a single value, accepting only the lower-bound range value as an order dose/rate input despite the original physician order for 1-10 Units/hr. Consequently, it marked all of the MAR dose/rate values as causing discrepancies in this single patient. This is a technical limitation of the system design. If the system had been able to accommodate dosing ranges in orders, it should have analyzed the MARs appropriately and avoided false-positive alerts.

**Figure 6:**
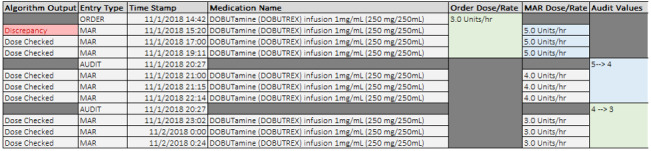
Example of an “order/audit value overwriting” issue leading to false positive calls from the system. Due to an ETL process, the original order value is repeatedly overwritten by the newer order audit values and ends up with the value of the last order audit record. When compared to the MAR documentations (which are correct), the false value in the order causes the algorithms to ‘detect’ a discrepancy, which is a false positive. ETL: extract, transform, load; MAR: medication administration record.

The second cause of deviation is related to an issue where original order doses/rates were overwritten or replaced by each new audit value, a consequence of the data extraction, transforming, and loading operations of the EHR software. We previously reported on this phenomenon in detail; it is the result of how the proprietary EHR system updates and stores audited order values in the retrospective database [22]. Figure 6 presents an example of this phenomenon. The original order value should be “5.0 Units/hr” (as evidenced by the first audit that changed dose from 5 to 4) but was listed as “3.0 Units/hr” that reflected the last dose modification (the second audit). Consequently, the first MAR was marked as discrepant. This issue resulted in inflated discrepancy rates because the first MAR could always be marked as discrepant if the original order value was no longer presented in our data. This data extraction, transforming, and loading pattern was confirmed by the team’s suspicions upon inspecting order values in the real-time production EHR system and comparing them to the retrospective data extracts. Astute readers may also notice that only the first MAR was considered discrepant by the system in Figure 6. This is because the system implements a “check the value with previous MAR data” logic that overrides subsequent discrepancy calls when the MAR values do not change in order to avoid overcalling discrepancies. As such, the first is considered a discrepancy, while subsequent consecutive MARs do not trigger a discrepancy to be called, by design.

**Figure 7:**
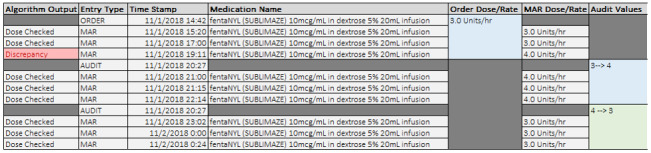
Example of the delayed entry of a verbal order causing a discrepancy to be detected. A verbal order was given at the bedside and the medication was appropriately adjusted, but the order was not documented until after the MAR documentation was placed. The algorithms allow a 30-minute window for verbal orders to be entered before calling a discrepancy, but in this example the order audit for the verbal order rate was not entered until 76 minutes later. MAR: medication administration record.

Lastly, there were discrepancies associated with changes to dosage (manifested as MAR documentations) that occurred greater than 30 minutes before the order was entered into the EHR. Such might occur as a result of an emergency during which a verbal order at the bedside is performed but not timely documented in the EHR. As such, the system implemented a 30-minute time window to account for these known lags in documentation due to verbal ordering while meeting the institutional expectations. This phenomenon is depicted in Figure 7, where the rate was changed to “4.0 Units/hr” 76 minutes before the order was modified. By reviewing the patient chart, we confirmed that the dose was changed via a verbal order and the administration was correct. However, the system marked the corresponding MAR as a discrepancy given that there was no audit or new order entered into the EHR for over 30 minutes after the administration. As a quick sensitivity analysis, we modified the algorithms to accept orders within a 60-minute time window; a comparison of discrepancy rates demonstrated a minimal impact, with rates changing less than 0.142% across all medications.

### Phase 4: Suggested Customization of the System or Clinical Workflows to Enable Better Detection of Medication Administration Errors

The system found discrepancies in medication administration that were attributed to both technical and clinical factors, which contributed to the initial poor fit of discrepancy detection on some medications at the implementation site (WFBMC). To overcome these barriers to successful implementation, the algorithms should be customized to adapt to the local institution. As an initiative, we customized the algorithms with a software update to solve 1 of the 3 major sources of false-positive discrepancies: order/audit value overwrites (the second issue identified in phase 3).

**Figure 8:**
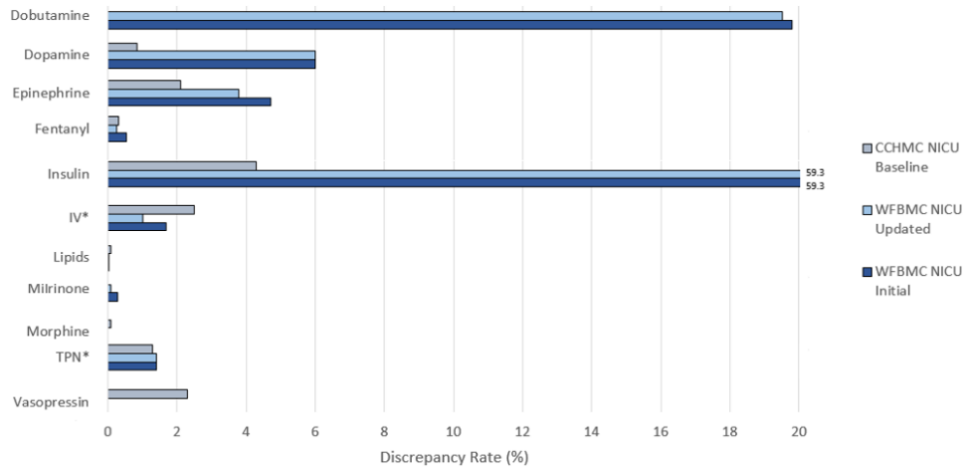
A comparison of discrepancy rates between (A) CCHMC NICU, (B) WFBMC NICU using the updated MED.Safe, and (C) WFBMC using the original MED.Safe. CCHMC: Cincinnati Children’s Hospital Medical Center; IV: intravenous; NICU: neonatal intensive care unit; TPN: total parenteral nutrition; WFBMC: Wake Forest Baptist Medical Center.

The investigative team (all study authors) implemented a patch to MED.Safe to recover the original order values from the sequences of medication use data. We then re-executed the updated system on the data used in the initial analysis to study its effects. Figure 8 and Table 3 demonstrate its effects in decreasing the output discrepancy rates for fentanyl, dobutamine, epinephrine, milrinone, and IV fluids. The other medications retained their discrepancy rates prior to the update, implying that they were not affected by order/audit value overwriting errors. As a result of this update, discrepancy rates from the WFBMC NICU became comparable to those from the CCHMC NICU for 5 of 9 medications with orders. The remaining medications maintained rates approximately twofold higher than the baseline CCHMC rates. Although this customization corrected for order/audit value overwriting errors, false-positive discrepancies persist as a result of delayed documentation of verbal orders and dosing range issues.

**Table 3:** Discrepancy rates of medication administration in the NICU before and after implementation of a software update at WFBMC in comparison to the site of development CCHMC.

Medication	*Initial* discrepancy rates in WFBMC^a^ NICU^b^, %	*Updated* discrepancy rates in WFBMC NICU, %	Absolute change in discrepancy rate, %	Initial discrepancy rates in CCHMC^c^ NICU, %
Dobutamine	19.8	19.5	–0.3	0.0
Dopamine	6.0	6.0	0.0	0.9
Epinephrine	4.7	3.8	–0.9	2.1
Fentanyl	0.5	0.25	–0.25	0.3
Insulin	59.3	59.3	0.0	4.3
Intravenous fluids	1.7	1.0	–0.7	2.5
Lipids	0.0	0.0	0.0	0.1
Milrinone	0.3	0.19	–0.11	0.0
Morphine	N/A^d^	N/A	N/A	0.1
Total parenteral nutrition	1.4	1.4	0.0	1.3
Vasopressin	N/A	N/A	N/A	2.3

^a^WFBMC: Wake Forest Baptist Medical Center.

^b^NICU: neonatal intensive care unit.

^c^CCHMC: Cincinnati Children’s Hospital Medical Center.

^d^N/A: not applicable. In 2018, no orders for continuous morphine or vasopressin were placed in the WFBMC NICU.

## Discussion

### Principal Findings

The ability to effectively implement the MED.Safe package at a second site is the first critical step toward creating a scalable and impactful solution for detecting and mitigating medication errors. This study investigated the feasibility and success of implementation for MED.Safe at a second site distinct from the origin of the software. The system outputs, such as descriptive statistics from local EHR data and discrepancy rates, served as a means to understand the institutional clinical workflows and prescribing patterns, assess the system generalizability, and help develop site-specific customizations. It is our hope that this study will serve as a guide for future institutions to efficiently assess the applicability of MED.Safe and lead to its implementation in an effort that maximizes medication safety in clinical settings.

Consideration of the clinical policies and workflows surrounding medication ordering, auditing, and MARs was vital in determining the feasibility of MED.Safe implementation at WFBMC. We observed that the NICU, PICU, and adult medical ICU were fundamentally different in their prescribing and auditing patterns (Table 1 and Multimedia Appendices 1-5). The WFBMC NICU had the most orders, audits, and MARs for the studied medications, reflecting the fact that MED.Safe was originally designed for an NICU setting that did not include common adult vasopressors such as norepinephrine. The adult medical ICUs had far less medication orders despite greater bed count. This was partially due to the fact that norepinephrine would have contributed 1466 orders to the total order count in this environment if an algorithm was available in MED.Safe to detect discrepancies; if included, the descriptive statistics would have more closely correlated with the bed count across the units. Regardless, the descriptive statistics output by the system allowed us to quickly understand, at the aggregate level, how prevalent the medications and MAR documentations were in different clinical environments and where the system may be the most useful. For instance, we found from the descriptive statistics that the NICU did not have vasopressin and morphine orders. As such, the algorithms for those medications not prescribed would not have any utility in the NICU and implementing MED.Safe there would yield no benefit. Beyond the basic descriptive characteristics, the comparison between audit/order ratios at WFBMC and CCHMC (Figure 2 and Multimedia Appendices 1-5) allowed us to understand the differences in prescribing workflows between the institutions. The lower audit/order ratios at WFBMC in comparison to CCHMC lead us to believe that WFBMC tends to create new orders for medication dose/rate changes, whereas CCHMC modifies existing orders for such changes more frequently. The more frequent use of order dose range intervals in combination with practices of documenting MAR rate to verify values very frequently may have contributed to the higher MAR/(order + audit) ratios at the WFBMC NICU despite fewer orders and audits overall (compared to CCHMC NICU). Our findings highlight potential practice differences across institutions, which may change the distribution of discrepancy rates, introduce additional opportunities to identify errors, or suggest the need for customizations to the MED.Safe system.

In phase 2, we executed the discrepancy detection algorithms of the software and analyzed the output discrepancy rates at WFBMC (Table 2). The rates at WFBMC aligned well with the ones at CCHMC for the majority of the studied medications. However, the rates at WFBMC varied widely, ranging from 0% to 59%, compared to CCHMC rates that ranged from 0% to 4.3%. The results suggested that the algorithms generalized well to the data and clinical practices for some medications but fit poorly for the others. Further inspection for the poorly performing medications in phase 3 identified 3 phenomena that contributed to the inflated discrepancy rates: range-based dosing, order/audit value overwriting in the data, and verbal ordering practices.

WFBMC uses dosing ranges to allow for bedside adjustment of a medication so long as the dosing is in range of the order and follows ancillary instructions, protocols, or policies. Such practice is common in adult medication prescribing, particularly in the administration of insulin, where dosing might shift within a given range depending on the trend of blood glucose values or intake of food. However, the algorithms were not equipped to deal with ordering ranges because at CCHMC site-specific practices required that an order dose/rate should be determinate and an audit (modification) be documented each time a dose/rate was changed. Consequently, WFBMC had comparatively fewer audits and more discrepancies for values within the acceptable dosing range. This difference in site-specific practices resulted in high discrepancy rates for insulin (59.3% at WFBMC NICU versus 4.3% at CCHMC NICU). A quick glance at the descriptive data and discrepancy rates generated by the algorithms will cue future customizations as to the cause of the high rates and shortcut much of the time spent in exploration and validation.

Second, the investigative team (all study authors) determined that the institutional EHR was overwriting the original order values with each new audit. The overwriting resulted in a notable amount of false-positive discrepancies on the first MARs. We were able to overcome this EHR-derived technical limitation with a software update that recovered the original order dose/rate by reasoning through from the sequences of order-audit data.

Lastly, a portion of discrepancies originated from dose/rate changes with delayed order documentation. This often occurs in emergency settings where verbal orders are first placed, while electronic orderings are documented after the care is delivered. The “grace period” for entering the electronic orders varies between institutions based on the site-specific clinical practices. Operating under verbal orders without proper documentation and procedure is high risk, and it creates a blind spot for errors that may have occurred but lacked the appropriate data for the system to detect them. The inability to identify medication errors during this elapsed time might lead to perpetuation of similar errors for an extended period, ultimately lessening the value of the system in identifying errors efficiently. A change in policy to eliminate the practice of verbal ordering is one potential solution, but this does not fit with the reality of clinical practice. Another solution is to adapt the system to the “grace period” that complies with local policies surrounding verbal ordering. For instance, the MED.Safe algorithms adopted a period of 30 minutes given the institutional expectations at CCHMC, which could be extended to 45-60 minutes to comply with WFBMC’s verbal ordering policies. In our quick sensitivity analysis we found that an extension to a 60-minute window, however, did not greatly reduce the discrepancy rate. This effect appears to be site specific as we have seen this change decrease rates to a greater degree at other sites. In the future, we will add this customizable feature to the software so that the grace period can be adjusted depending on the care setting and local policy. This will also allow an automated version of the sensitivity analysis. Ultimately, the system could be more flexible and customizable to fit each institution and even department that varies in health care policy and procedures surrounding the medication use life cycle.

In phase 4, we addressed the order/audit value overwriting issue through a software update. It reduced false-positive discrepancies output by the system for most of the studied medications. The remaining 2 medications (dobutamine and insulin) with discrepancy rates notably higher than baseline CCHMC rates are largely due to the range-based dosing issue. Further reduction in false-positive discrepancies can therefore be obtained by addressing the other 2 issues, range-based dosing and verbal ordering practices. Efforts to do so are planned for future work.

Our study suggested that it was feasible to implement MED.Safe in a setting external to the development environment. However, the software package did not account for all the differences in medication administration practices at the implementation site, with a resultant impact on its performance. The identified barriers to proper fitting of the system can be overcome through both clinical practice change/policy reform and the addition of algorithm customizations where appropriate. We were able to identify targets for algorithm customization to account for these practices and to address one of those issues efficiently. These efforts have greatly advanced our knowledge of the portability of the MED.Safe and have shown us what work is left to do in order to further improve its generalizability.

### Conclusions

The implementation of the MED.Safe system at a second site was a feasible and efficient way to track medical administration discrepancies. Analysis of medication use data and discrepancy rates output by the system revealed local medication prescribing patterns, and comparison against implementation at the original site suggested areas of both good and poor fit. Overall fit was enhanced through the implementation of a software update. To maximize efficiency in accurately detecting and correcting medication errors, modifications must be made to both the MED.Safe software package and suboptimal clinical practices. Such modifications should increase the system’s customizability to the local clinical workflows and policies, ultimately improving its accuracy and generalization for external use.

## Appendix

Multimedia Appendix 1 A comparison of medication ordering, auditing, and MAR data by drug generated by MED.Safe at Wake Forest Baptist Medical Center. MAR: medication administration record.

Multimedia Appendix 2 A comparison of medication ordering, auditing, and MAR data by generated by MED.Safe at Wake Forest Baptist Medical Center. MAR: medication administration record.

Multimedia Appendix 3 Descriptive statistics of orders, audits, and medication administration record data at all Wake Forest Baptist Health ICUs during the study period. ICU: intensive care unit.

Multimedia Appendix 4 Descriptive statistics of orders, audits, and medication administration record data in the Wake Forest Baptist Health NICU during the study period. NICU: neonatal intensive care unit.

Multimedia Appendix 5 Descriptive statistics of orders, audits, and medication administration record data in the Cincinnati Children’s Hospital NICU during the study period. NICU: neonatal intensive care unit.

## Abbreviations

CCHMC: Cincinnati Children’s Hospital Medical Center
EHR: electronic health record
ICU: intensive care unit
IV: intravenous
MAE: medication administration error
MAR: medication administration record
NICU: neonatal intensive care unit
PICU: pediatric intensive care unit
TPN: total parenteral nutrition
WFBMC: Wake Forest Baptist Medical Center
