# Supplementary material for: The Generalizability of a Medication Administration Discrepancy Detection System: Quantitative Comparative Analysis
Source: JMIR Med Inform. 2020 Dec 2;8(12):e22031. doi: 10.2196/22031 (PMC7744260; doi:10.2196/22031)
Supplement: Multimedia Appendix 1 [file medinform_v8i12e22031_app1.docx]

| **Drug** | **Department** | **Orders** | **Audits** | **MARs** |
| --- | --- | --- | --- | --- |
|  | Neonatal Intensive Care Unit (NICU) | 16 | 44 | 232 |
| Epinephrine | Pediatric Intensive Care Unit (PICU) | 69 | 110 | 2,667 |
|  | ICU Medicine Unit | 9 | 9 | 161 |
|  | Neonatal Intensive Care Unit (NICU) | 106 | 90 | 5069 |
| Dopamine | Pediatric Intensive Care Unit (PICU) | 14 | 24 | 275 |
|  | ICU Medicine Unit | 1 | 1 | 9 |
|  | Neonatal Intensive Care Unit (NICU) | 37 | 52 | 1,710 |
| Dobutamine | Pediatric Intensive Care Unit (PICU) | 2 | 3 | 15 |
|  | ICU Medicine Unit | 19 | 15 | 615 |
| Insulin | Neonatal Intensive Care Unit (NICU) | 7 | 6 | 86 |
|  | Pediatric Intensive Care Unit (PICU) | 30 | 104 | 1,015 |
|  | ICU Medicine Unit | 106 | 64 | 3,132 |
| IV | Neonatal Intensive Care Unit (NICU) | 466 | 356 | 16,333 |
|  | Pediatric Intensive Care Unit (PICU) | 719 | 371 | 20,760 |
|  | ICU Medicine Unit | 1,301 | 358 | 18,742 |
|  | Neonatal Intensive Care Unit (NICU) | 2,968 | 243 | 64,609 |
| TPN | Pediatric Intensive Care Unit (PICU) | 486 | 13 | 10,549 |
|  | ICU Medicine Unit | 57 | 0 | 947 |
|  | Neonatal Intensive Care Unit (NICU) | 0 | 0 | 0 |
| Morphine | Pediatric Intensive Care Unit (PICU) | 90 | 115 | 7,232 |
|  | ICU Medicine Unit | 79 | 33 | 813 |
|  | Neonatal Intensive Care Unit (NICU) | 0 | 0 | 0 |
| Vasopressin | Pediatric Intensive Care Unit (PICU) | 20 | 38 | 781 |
|  | ICU Medicine Unit | 172 | 31 | 4,039 |
|  | Neonatal Intensive Care Unit (NICU) | 200 | 312 | 18,312 |
| Fentanyl | Pediatric Intensive Care Unit (PICU) | 63 | 81 | 4,035 |
|  | ICU Medicine Unit | 184 | 50 | 9,399 |
|  | Neonatal Intensive Care Unit (NICU) | 24 | 25 | 5,283 |
| Milrinone | Pediatric Intensive Care Unit (PICU) | 44 | 67 | 7,008 |
|  | ICU Medicine Unit | 17 | 15 | 829 |
|  | Neonatal Intensive Care Unit (NICU) | 2,566 | 9 | 55,245 |
| Lipids | Pediatric Intensive Care Unit (PICU) | 428 | 8 | 8,443 |
|  | ICU Medicine Unit | 5 | 0 | 101 |
