# Supplementary material for: The Generalizability of a Medication Administration Discrepancy Detection System: Quantitative Comparative Analysis
Source: JMIR Med Inform. 2020 Dec 2;8(12):e22031. doi: 10.2196/22031 (PMC7744260; doi:10.2196/22031)
Supplement: Multimedia Appendix 2 [file medinform_v8i12e22031_app2.docx]

| **Department** | **Drug** | **Order** | **Audit** | **MAR** |
| --- | --- | --- | --- | --- |
| Neonatal Intensive Care Unit (NICU) | Epinephrine | 16 | 44 | 232 |
|  | Dopamine | 106 | 90 | 5,069 |
|  | Dobutamine | 37 | 52 | 1,710 |
|  | Insulin | 7 | 6 | 86 |
|  | IV | 466 | 356 | 16,333 |
|  | TPN | 2,968 | 243 | 64,609 |
|  | Morphine | 0 | 0 | 0 |
|  | Vasopressin | 0 | 0 | 0 |
|  | Fentanyl | 200 | 312 | 18,312 |
|  | Lipids | 2,566 | 9 | 55,245 |
|  | Milrinone | 24 | 25 | 5,283 |
| Pediatric Intensive Care Unit (PICU) | Epinephrine | 69 | 110 | 2,667 |
|  | Dopamine | 14 | 24 | 275 |
|  | Dobutamine | 2 | 3 | 15 |
|  | Insulin | 30 | 104 | 1,015 |
|  | IV | 719 | 371 | 20,760 |
|  | TPN | 486 | 13 | 10,549 |
|  | Morphine | 90 | 115 | 7,232 |
|  | Vasopressin | 20 | 38 | 781 |
|  | Fentanyl | 63 | 81 | 4,035 |
|  | Lipids | 428 | 8 | 8,443 |
|  | Milrinone | 44 | 67 | 7,008 |
| ICU Medicine Unit | Epinephrine | 9 | 9 | 161 |
|  | Dopamine | 1 | 1 | 9 |
|  | Dobutamine | 19 | 15 | 615 |
|  | Insulin | 106 | 64 | 3,132 |
|  | IV | 1,301 | 358 | 18,742 |
|  | TPN | 57 | 0 | 947 |
|  | Morphine | 79 | 33 | 813 |
|  | Vasopressin | 172 | 31 | 4,039 |
|  | Fentanyl | 184 | 50 | 9,399 |
|  | Lipids | 5 | 0 | 101 |
|  | Milrinone | 17 | 15 | 829 |
