# Supplementary material for: The Generalizability of a Medication Administration Discrepancy Detection System: Quantitative Comparative Analysis
Source: JMIR Med Inform. 2020 Dec 2;8(12):e22031. doi: 10.2196/22031 (PMC7744260; doi:10.2196/22031)
Supplement: Multimedia Appendix 3 [file medinform_v8i12e22031_app3.docx]

| **Drug/Process** | **Audit** | **Order** | **Audit/Order** | **MAR/(Order+Audit)** |
| --- | --- | --- | --- | --- |
| Dobutamine | 186 | 151 | 1.2 | 17.1 |
| Dopamine | 150 | 142 | 1.1 | 20.1 |
| Epinephrine | 393 | 396 | 1.0 | 15.1 |
| Fentanyl | 545 | 662 | 0.8 | 32.7 |
| Insulin | 288 | 915 | 0.3 | 16.5 |
| IV | 2797 | 8477 | 0.3 | 18.8 |
| Lipid | 24 | 3012 | 0.0 | 21.1 |
| Milrinone | 381 | 374 | 1.0 | 37.5 |
| Morphine | 174 | 283 | 0.6 | 19.5 |
| TPN | 256 | 3874 | 0.1 | 20.0 |
| Vasopressin | 177 | 623 | 0.3 | 22.1 |
