# Supplementary material for: The Generalizability of a Medication Administration Discrepancy Detection System: Quantitative Comparative Analysis
Source: JMIR Med Inform. 2020 Dec 2;8(12):e22031. doi: 10.2196/22031 (PMC7744260; doi:10.2196/22031)
Supplement: Multimedia Appendix 4 [file medinform_v8i12e22031_app4.docx]

| **Drug/Process** | **Order** | **Audit** | **Audit/Order** | **MAR/(Order+Audit)** |
| --- | --- | --- | --- | --- |
| Dobutamine | 37 | 52 | 1.5 | 19.2 |
| Dopamine | 106 | 90 | 0.9 | 25.9 |
| Epinephrine | 16 | 44 | 2.8 | 2.9 |
| Fentanyl | 200 | 312 | 1.6 | 35.8 |
| Insulin | 7 | 6 | 0.9 | 6.6 |
| IV | 466 | 356 | 0.8 | 19.9 |
| Lipid | 2,566 | 9 | 0.0 | 21.5 |
| Milrinone | 24 | 25 | 1.0 | 107.8 |
| Morphine | 0 | 0 | 0.0 | 0.0 |
| TPN | 2,968 | 243 | 0.1 | 20.1 |
| Vasopressin | 0 | 0 | 0.0 | 0.0 |
