# Supplementary material for: The Generalizability of a Medication Administration Discrepancy Detection System: Quantitative Comparative Analysis
Source: JMIR Med Inform. 2020 Dec 2;8(12):e22031. doi: 10.2196/22031 (PMC7744260; doi:10.2196/22031)
Supplement: Multimedia Appendix 5 [file medinform_v8i12e22031_app5.docx]

| **Drug/Process** | **Audit** | **Order** | **Audit/Order** | **MAR/(Order+Audit)** |
| --- | --- | --- | --- | --- |
| Dobutamine | 2 | 1 | 2.0 | 1.3 |
| Dopamine | 124 | 41 | 3.0 | 1.4 |
| Epinephrine | 684 | 151 | 4.5 | 1.9 |
| Fentanyl | 150 | 106 | 1.4 | 6.1 |
| Insulin | 103 | 30 | 3.4 | 1.2 |
| IV | 2809 | 2511 | 1.1 | 3.3 |
| Lipid | 16 | 4809 | 0.0 | 3.3 |
| Milrinone | 3 | 12 | 0.3 | 19.0 |
| Morphine | 340 | 241 | 1.4 | 5.7 |
| TPN | 5 | 4659 | 0.0 | 3.4 |
| Vasopressin | 150 | 42 | 3.6 | 1.6 |
